# Hot and Cold Baths in Alternation

**Published:** 1834

**Authors:** 


### IX.—Efficacy of the Hot and Cold bath in alternation, illustrated with a Case.

We are disposed to think, that the powers of the hot and cold bath, have not been sufficiently tested, in chronic diseases, unattended with obvious inflammatory action. In many cases of this kind, the skin is exanguinous, unperspiring, defective in temperature, and blunted in sensibility. In this pathological condition, we know of no cutaneous excitant so powerful, as the means just mentioned; and when the viscera of the abdomen are in a torpid condition, or the general nervous system is impaired in its sensibility, the excitement of the surface soon revives that of the parts beneath. The following case will serve to show, what may sometimes be accomplished by this simple application.

Case.—A gentleman of the Bar, at the age of 19 or 20, had an attack of gonorrhœa, which was followed for sometime by nocturnal emissions, which, however, had become much less frequent when he applied to us, at the age of 23. For a long time, however, he had labored under irregular bowels, being either costive or relaxed, and greatly afflicted with flatulence; his alvine discharges were often acrid, and sometimes of a green, but commonly of a light yellow color. His appetite was constantly craving; but the indulgence of it generally increased the flatulence, and occasionally excited pyrosis. His muscular system of animal life, became enfeebled and agitated, his heart suffered from palpitations, his spirits were greatly depressed, and he sought solitude.

In November, 1833, he discovered, that between the shoulder blades, and just above the sacrum, he had pain on pressure; which extended down the insides of the thighs while the outsides felt numb, that the flatulence of his bowels was at the same time augmented, and that cramps occurred in his legs and feet. Blisters applied to the upper part of the spine raised his spirits; to the lower part, were followed by flatulence, diarrhœa, and sometimes a considerable degree of fever.

He had previously to the time just mentioned, dieted himself, and taken the blue pill and rhubarb, with very little effect. After discovering the tenderness of the spine, he was cupped more than a dozen times, and also blistered, by a respectable young physician of Ohio. For a while this method afforded great relief, but at length did no further good; and when he applied to us his spine was still tender and painful under pressure, his bowels more or less irregular, digestion bad, hands perpetually cold, muscular system enfeebled, nervous functions generally, impaired, and his spirits gloomy.

We directed no medicines, but ordered the hot and cold bath, in immediate succession, every day at 11 o'clock, and every night before bedtime, with hard frictions to the skin. After even the first bathing he felt better, and in little more than a fortnight, was so well as to leave the city. He had regular and rather copious alvine evacuations without pain; the tenderness of his spine had greatly diminished; he slept better; had less nervous irritability and gloom; his hands were warm; his complexion improved, and his flesh and strength had greatly increased.

---

It is remarkable, that so many persons should have a dread of the alternation of hot and cold water to the skin. It signifies little, we suppose, which of them may be applied first, the effect in either case is to revive its sensibility, fill it with blood, and restore the perspiration.

The reason for employing a bath in the forenoon may not be very obvious, but we are persuaded it is, on the whole, the most proper period in the 24 hours. There seems to be at that time, between 11 and 1 o'clock, a diurnal failure, to some extent, of the powers of organic life, and an especial faultering of the calorific function. Hence, we find, that the cold stages of hectic and intermittent fevers, more frequently recur at that period, than any other, and dyspeptics are more liable to cold feet at that time than any other, in the day or night. The custom of taking a luncheon and some stimulating drink about that hour, probably had its origin, in the recurrence of a sinking sensation in the epigastrium, which people in general, and particularly the infirm, experience at that period more frequently than any other.

